# Neuro-Cells Mitigate Amyloid Plaque Formation and Behavioral Deficits in the APPswe/PS1dE9 Model of Alzheimer Disease While Also Reducing IL-6 Production in Human Monocytes

**DOI:** 10.3390/cells14151168

**Published:** 2025-07-29

**Authors:** Johannes de Munter, Kirill Chaprov, Ekkehard Lang, Kseniia Sitdikova, Erik Ch. Wolters, Evgeniy Svirin, Aliya Kassenova, Andrey Tsoy, Boris W. Kramer, Sholpan Askarova, Careen A. Schroeter, Daniel C. Anthony, Tatyana Strekalova

**Affiliations:** 1 Department of Psychiatry and Neuropsychology, Maastricht University, 6229 ER Maastricht, The Netherlands; h.demunter@neuroplast.com (J.d.M.); t.strekalova@pharm.ox.ac.uk (T.S.); 2 Neuroplast B.V., 6222 NK Maastricht, The Netherlands; chaprov@ipac.ac.ru (K.C.); e.lang@neuroplast.com (E.L.); svirin_ep@niiopp.ru (E.S.); bkramer@ump.edu.pl (B.W.K.); 3 Center for Life Sciences, National Laboratory Astana, Nazarbayev University, Astana 010000, Kazakhstan; sitdikova.k@niiopp.ru (K.S.); aliya.kassenova@nu.edu.kz (A.K.); andrey.tsoy@nu.edu.kz (A.T.); shaskarova@nu.edu.kz (S.A.); 4 Department of Neurology, University of Zurich, 8091 Zurich, Switzerland; ech.wolters@gmail.com; 5 Department of Neonatology, Poznan University of Medical Sciences, 60806 Poznan, Poland; 6 Preventive and Environmental Medicine, Kastanienhof Clinic, 50858 Cologne-Junkersdorf, Germany; 7 Department of Pharmacology, University of Oxford, Oxford OX1 3QT, UK; daniel.anthony@pharm.ox.ac.uk

**Keywords:** Alzheimer’s disease, APPswe/PS1dE9 mice, cytokines, stem cell therapy, neuroinflammation, amyloid plaques, emotionality, cognition

## Abstract

Neuroinflammation is a key feature of Alzheimer’s disease (AD), and stem cell therapies have emerged as promising candidates due to their immunomodulatory properties. Neuro-Cells (NC), a combination of unmodified mesenchymal stem cells (MSCs) and hematopoietic stem cells (HSCs), have demonstrated therapeutic potential in models of central nervous system (CNS) injury and neurodegeneration. Here, we studied the effects of NC in APPswe/PS1dE9 mice, an AD mouse model. Twelve-month-old APPswe/PS1dE9 mice or their wild-type littermates were injected with NC or vehicle into the cisterna magna. Five to six weeks post-injection, cognitive, locomotor, and emotional behaviors were assessed. The brain was stained for amyloid plaque density using Congo red, and for astrogliosis using DAPI and GFAP staining. Gene expression of immune activation markers (*Il-1β*, *Il-6*, *Cd45*, *Tnf*) and plasticity markers (*Tubβ3*, *Bace1*, *Trem2*, *Stat3*) was examined in the prefrontal cortex. IL-6 secretion was measured in cultured human monocytes following endotoxin challenge and NC treatment. Untreated APPswe/PS1dE9 mice displayed impaired learning in the conditioned taste aversion test, reduced object exploration, and anxiety-like behavior, which were improved in the NC-treated mutants. NC treatment normalized the expression of several immune and plasticity markers and reduced the density of GFAP-positive cells in the hippocampus and thalamus. NC treatment decreased amyloid plaque density in the hippocampus and thalamus, targeting plaques of <100 μm^2^. Additionally, NC treatment suppressed IL-6 secretion by human monocytes. Thus, NC treatment alleviated behavioral deficits and reduced amyloid plaque formation in APPswe/PS1dE9 mice, likely via anti-inflammatory mechanisms. The reduction in IL-6 production in human monocytes further supports the potential of NC therapy for the treatment of AD.

## 1. Introduction

Alzheimer’s disease (AD) is a progressive neurodegenerative disorder and the most common cause of dementia in the elderly. It is characterized by neuronal loss, cognitive decline, emotional disturbances, and functional impairment [1]. Currently, no disease-modifying therapies are available, and symptomatic treatments offer limited benefits.

Evidence increasingly supports a role for neuroinflammation in AD pathophysiology, prompting interest in anti-inflammatory interventions. The accumulation of amyloid beta (Aβ) oligomers is linked to the over-production of pro-inflammatory cytokines [2,3,4]. In AD patients, elevated plasma IL-6 correlates with cognitive decline [5,6], and brain overexpression of IL-1β facilitates leukocyte infiltration and neurodegeneration [7,8]. Neutralizing IL-6 in the brain of APPswe/PS1dE9 mice alleviated memory deficits [9]. Stem cell therapies have also shown the potential to suppress IL-6 levels in both CNS and periphery in clinical and preclinical studies [10,11,12,13,14,15,16].

IL-6 overexpression activates the JAK/STAT3 pathway, a key inflammatory signaling cascade involved in microtubule regulation via α- and β-tubulin gene expression [17,18,19,20,21]. Upon IL-6 binding to its receptor complex (IL-6R and gp130), associated Janus kinases (JAKs) phosphorylate STAT3 at Tyr705, a step essential for its dimerization and nuclear translocation [1,2,3]. Once activated, STAT3 regulates transcription of genes such as *Bcl-2*, *Cyclin D1*, *c-Myc*, and *Socs3*, which influence cell survival and proliferation [4]. Chronic activation of STAT3 is implicated in immune dysregulation and neuroinflammatory responses [5]. In the APPswe/PS1dE9 model, STAT3 has been associated with cognitive decline [2,22] and is involved in modulating astroglial responses around Aβ plaques, contributing to their clearance [23,24].

Astrocytic activation by Aβ aggregates is another contributor to AD-associated neuroinflammation, promoting tau hyperphosphorylation and intracellular deposition [25,26,27]. Aβ exposure upregulates GFAP and pro-inflammatory cytokines, such as IL-1β, IL-6, and TNF [28,29,30], activating neuronal pathways, including p38 MAPK, JNK, and GSK-3β, which drive pathological tau phosphorylation [31,32]. This inflammatory cascade also disrupts glutamate homeostasis, enhances oxidative stress, and impairs mitochondrial function, further accelerating tau pathology [33]. The result is a feedforward loop where astrocyte-driven inflammation exacerbates both Aβ and tau pathologies, worsening neuronal damage and cognitive decline.

Despite these insights, conventional anti-inflammatory therapies have shown limited success in AD [34,35]. Stem cell-based approaches, particularly those involving mesenchymal stem cells (MSCs), have gained attention for their immunomodulatory properties [36]. MSCs are recruited to sites of inflammation and injury [37,38], where they exert pro-regenerative effects through paracrine signaling. They secrete a range of neurotrophic and angiogenic factors, extracellular vesicles, and biologically active molecules that modulate inflammation and support tissue repair via mechanisms such as α7 nicotinic acetylcholine receptor activation [37,38,39]. Recent studies using intravenously administered bone marrow-derived MSCs in AD mouse models confirmed therapeutic effects via β-amyloid PET imaging, behavioral testing, and histopathological assessment [36,39,40]. These cells secrete anti-inflammatory cytokines and growth factors, such as IL-10, VEGF, IGF-1, HGF, BDNF, and NGF [41,42], with secretion being enhanced under inflammatory conditions. A double-blind, phase I clinical trial of Lomecel-B, an allogeneic bone marrow MSC preparation, reported improved cognitive outcomes and reductions in serum IL-6, alongside increased IL-4 and IL-10 in 25 patients with mild AD [40].

Although most research has focused on MSCs, there is growing interest in the therapeutic potential of hematopoietic stem cells (HSCs) in neurodegenerative diseases. HSCs can differentiate into endothelial cells and may aid in repairing vascular dysfunctions, such as increased blood–brain barrier (BBB) permeability seen in AD. Bone marrow-derived endothelial precursors have been proposed as an AD therapy [43,44]. Combining MSCs with HSCs may enhance neuroprotective effects, yet few studies have compared these cell types directly in vivo. In the bone marrow niche, MSCs (CD105^+^, CD90^+^, CD271^+^, CD73^+^) maintain HSCs (CD34^+^) in an undifferentiated state; loss of this balance promotes cellular aging and death [45,46]. Consistent with this view, a series of studies have demonstrated beneficial effects of a combined bone marrow-derived MSC and HSC preparation Neuro-Cells (NC) in models of amyotrophic lateral sclerosis (ALS), frontotemporal dementia (FTD), and spinal cord injury [14,16,47]. Single intracerebroventricular injections of NC (1.39 × 10^6^ total cells, including 5 × 10^5^ CD34^+^ HSCs and MSCs expressing CD105, CD90, CD271, CD73) reduced IL-1β and IL-6 levels in serum and the lumbar part of spinal cord of FUS [1-359]-tg mice, exceeding the effects of celecoxib [14,16]. In spinal cord injury models, intrathecal NC administration lowered TNF, IL-1β, and IL-6 levels and improved behavioral outcomes [47].

Building on these findings, we examined the neuroprotective potential of NC in the APPswe/PS1dE9 (APP/PS1) transgenic mouse model of AD. These mice express mutations in APP and PSEN1/2, leading to enhanced β-secretase and γ-secretase activity and rapid Aβ accumulation [48]. APP/PS1 mice exhibit amyloid plaques, tau tangles, memory deficits, and hyperactivity [49,50]. Given the higher AD prevalence and greater cognitive impairment in women, we employed older female mutants to enhance clinical relevance and model validity.

In this study, we administered NC (containing human MSCs and CD34^+^ HSCs) or vehicle into the cisterna magna of 12-month-old female APP/PS1 mice and their wild-type (WT) littermates. Five to six weeks later, we assessed cognition, emotion, locomotion, and anxiety-related behaviors. To evaluate AD pathology, we quantified amyloid plaque density (Congo red) and astrocyte activation (GFAP/DAPI) in the hippocampus, thalamus, and cortex. We also measured prefrontal cortex expression of pro-inflammatory cytokines (*Il-1β*, *Tnf*, *Il-6*) and AD-relevant genes linked to regeneration and cellular plasticity (*Cd45*, *Stat3*, *Tubb3*, *Trem2*, *Bace1*). Finally, to evaluate systemic anti-inflammatory activity, we analyzed IL-6 secretion from human monocytes challenged with LPS.

## 2. Materials and Methods

### 2.1. Animals and Study Design

Twelve-month-old female APP/PS1 and WT littermates were bred on a C57BL/6 background and housed in groups of three to five per cage under a reversed 12 h light–dark cycle (lights on: 21:00) with free access to food and water (controlled laboratory conditions, 22 ± 1 °C, 55% humidity) [51]. All procedures were approved by the Local Ethics Committee PE “National Laboratory of Astana,” Nazarbayev University (20 March 2023, N02-2023) and complied with Directive 2010/63/EU and ARRIVE guidelines.

Female transgenic animals were selected, as they exhibit greater vulnerability to Alzheimer’s disease (AD) pathology compared to males. For example, female APP/PS1, 3xTg-AD, and Tg2576 mice develop earlier and more pronounced amyloid-β plaque accumulation, neuroinflammation, and cognitive deficits than age-matched males [52,53]. Epidemiological data also show that women have a higher lifetime risk of developing AD. Therefore, using female APP/PS1 mice provides greater construct validity for modeling the human condition [54].

**Figure 1:**
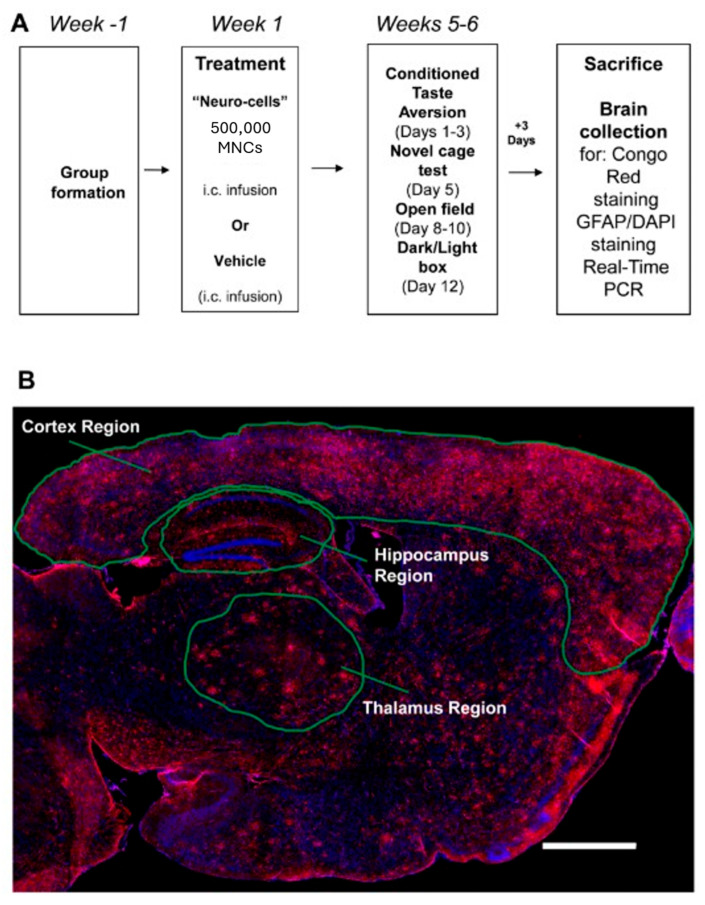
A study flow and anatomical regions investigated. (**A**) A study flow of NC administration, behavioral, histological and molecular assays. (**B**) Low power fluorescence (GFAP/DAPI) image showing the regions studied in the histological analysis of β-amyloid deposition and GFAP. Scale bar: 1000 μm.

**Figure 2:**
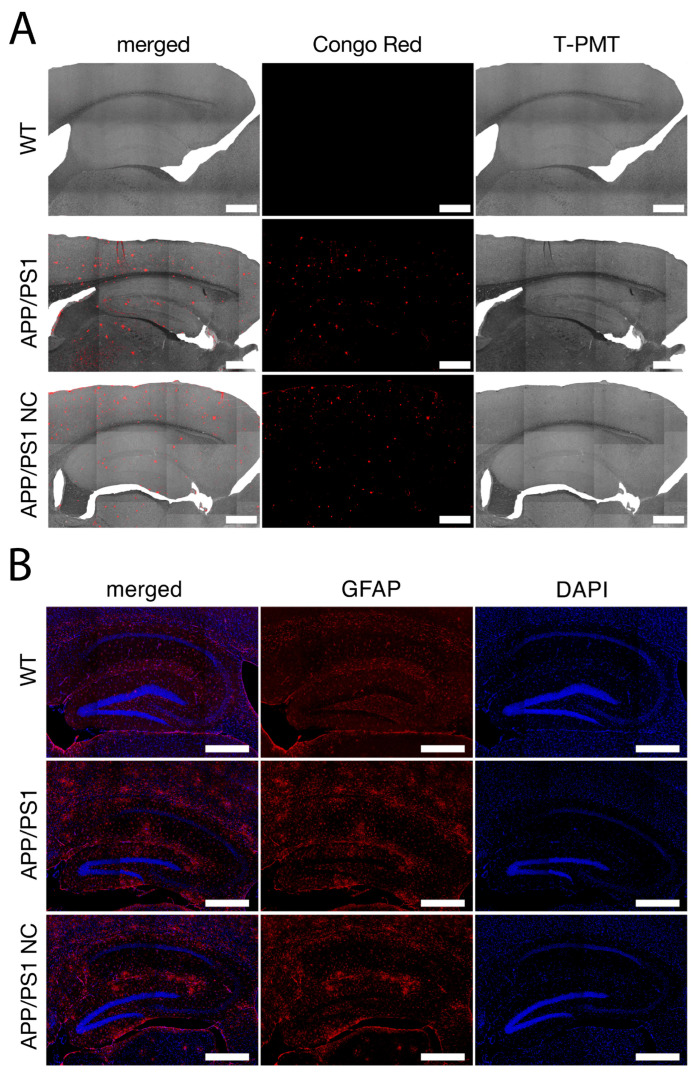
Representative images of the Congo red-stained amyloid plaques and astroglia. (**A**) Representative images of the mouse brains stained with Congo red of WT mice, non-treated APP/PS1, and APP/PS1-NC groups. Scale bar: 500 μm. (**B**) Representative images of hippocampal zone of mouse brain stained with anti-GFAP (red) and merged with DAPI staining. Scale bar: 500 μm.

In total, 16, 10, and 18 mice were used in the APP/PS1, APP/PS1-NC, and WT groups, respectively. APP/PS1 mice and WT mice were injected into the cisterna magna with a fresh NC preparation in Ringer solution or with Ringer solution alone (vehicle). As the previous central administration of an NC preparation or Ringer’s solution alone had no negative effects on animal physiology and behavior [14,16], and in compliance with the RRRs, WT-NC and APP/PS1-non-injected groups were not included in the present study. Body weights were monitored throughout the recovery period. At five–six weeks the mice were assessed using a battery of tests for learning, locomotion, and emotionality (see Figure 1A). All mice were subjected to the following behavioral tests: (1) conditioned taste aversion model (days 1–3), (2) novel cage exploration test (day 5), (3) open field locomotion test (days 8–10), and (4) dark–light box paradigm (day 12). Sufficient inter-test intervals were maintained to minimize stress in older animals [41]. Three days after the final behavioral assay, the mice were euthanized (Figure 1A; see below). The prefrontal cortex was collected from one hemisphere for RNA isolation, cDNA synthesis, and RT-PCR assay (see Section 2.6). The other hemisphere was used for histological analysis of amyloid plaque formation using Congo red staining (see Figure 1B), and for immunohistochemical analysis of astrogliosis with GFAP antibody and DAPI staining (see Figure 2A,B). Experimenters were blind to animals’ identity. No animals were excluded from the study.

### 2.2. Neuro-Cells Preparation

NC preparations (Neuroplast BV, Maastricht, Netherlands) contained 5 × 10^5^ total cells (MSCs and HSCs) per 10 µL dose. MSC marker expression was confirmed by FACS: CD105^+^ (85.6%), CD90^+^ (13%), CD271^+^ (7%), and CD73^+^ (4%). Cells were transported at 4 °C and injected within 36 h in Ringer’s solution as previously described [14,16]. Viability was checked 2 h prior to administration. Pilot studies, which were undertaken to determine the distribution of the infused Neuro-Cells and to optimize the protocols for i.c.v. administration, revealed the presence of the cells in the brain using immunohistochemical staining with human anti-mitochondrial antibodies 12 and 24 h after injection of 5 × 10^5^ cells [14].

### 2.3. Stereotaxic Surgery and Administration of Neuro-Cells

Animals were anesthetized with halothane and fixed in a stereotaxic frame (World Precision Instruments, Sarasota, TX, USA) for unilateral i.c. infusion via a burr hole made in the skulls of mice; coordinates: AP −1.1 mm (from Obex), ML +0.5 mm, DV −1.3 mm (from brainstem surface) as previously described [16]. Using an automated stereotaxic injector (RWD Life Science Co., Shenzhen, China), 10 µLNC or vehicle was infused into the cisterna magna over 10 min. Wounds were closed with Vicryl™ rapide 5.0 (Ethicon™, Somerville, NJ, USA) and animals were monitored post-operatively with analgesia for two days.

### 2.4. Behavioral Assays for Learning and Emotionality

#### 2.4.1. Conditioned Taste Aversion Learning

On day 0–1, during the training session, mice were deprived from water between 17.00 (day 0) and 14.00 (day 1) (for 21 h) and were then allowed to drink a 2.5% sucrose solution for 30 min in a one-bottle paradigm [55,56]. Following this exposure to concentrated sucrose solution, the mice received an intraperitoneal injection of solution of lithium chloride (LiCl, 0.24 M) at a dose of 2% of body weight or PBS. After the injection, the animals were allowed to have access to a sucrose solution for another 1.5 h and thereafter they were water deprived for 12 h. On day 2 of the assay, a test for memory recall was carried out. Animals were given a choice between tap water and 1% sucrose solution in a two-bottle paradigm for 8 h. The amount of water and sucrose solution consumed was determined by weighing the bottles before and after a drinking session, and the preference to sucrose solution was calculated according to the following formula: Sucrose preference = 100% × (Amount of sucrose solution consumed, g)/(Total amount of liquid consumed, g). A decrease in sucrose preference during the recall session in comparison with a chance level of 50% of drinking from either of the two bottles was considered a sign of taste aversion [55,56], i.e., inhibitory associative learning.

#### 2.4.2. Novel Cage

The novel cage test was performed to assess vertical exploration activity in a new environment. Mouse was placed in a clear plastic cage (14 × 21 × 27 cm) with a small amount of fresh litter under low light intensity (5 lux). The number of rears and latency to the first rear were counted during a 3-min period [57,58].

#### 2.4.3. Open Field Paradigm

The open field test was carried out in square box (45 × 45 × 45 cm) under low light intensity (5 lux). The animal was placed in center of the box and its movements were tracked for 20 min period with a digital camera placed above the arena. Mean velocity was scored using automated off-line analysis (ViewPoint, Civrieux, France) as described before [59]. Mice were studied for total distance moved and mean velocity in order to rule out possible effects of the mutation and surgery on general locomotion.

#### 2.4.4. Dark–Light Box

Mice were placed into the black compartment (15 × 20 × 25 cm) from which they could visit the illuminated compartment (30 × 20 × 25 cm, 5 lux). During a 5-min period, the latency of the first transition, time spent in the light compartment and the number of transitions between compartments were recorded as described before [57,60].

### 2.5. Tissue Collection and Brain Histology

Three days after the final behavioral assay, the mice were euthanized (Figure 1A). Animals were transcardially perfused with 10 mL ice-cold saline, with the heart still contracting to ensure effective circulation of the saline. The prefrontal cortex was collected from the left hemisphere for RNA isolation, as described before [61], followed by cDNA synthesis, and RT-PCR assay (see Section 2.6). The procedure was followed by 4% paraformaldehyde perfusion via left ventricle of the heart, the right hemisphere of the brain was removed, post-fixed in formaldehyde overnight as described before [62] and then embedded in paraffin. Paraffin-embedded tissue was sectioned at 8 µm. Sections were deparaffinized, rehydrated, and stained using standard protocols as described before [62].

#### 2.5.1. Congo Red Staining, Plaque Microscopy and Scoring

Sections were stained with 0.5% Congo red in 50% ethanol (5 min), differentiated with 0.2% KOH in 80% ethanol (3 min), and mounted with Immu-Mount™ (Thermo Fisher Scientific Inc., Kalamazoo, MI, USA) as described before [63]. Ten sections per animal were analyzed by confocal microscopy using Zeiss LSM880 (Carl Zeiss, Oberkochen, Germany). Plaque morphology in hippocampus, thalamus, and cortex was quantified using QuPath v0.4.3 machine learning classifier (Northern Ireland, Belfast, UK) as described before [64]. Plaques were categorized by size: <100 µm^2^, 100–200 µm^2^, >200 µm^2^ (Figure 1B and Figure 2A). The number of each type of plaque was calculated per mm^2^ in each of examined brain regions.

#### 2.5.2. Immunohistochemical Analysis of Astrocyte Activation

We performed immunohistochemical staining for the astrocyte antigen GFAP (Figure 2B). Slices were boiled in 10 mM citrate buffer (pH 6) for 12 min at 700 W in a microwave, washed for 5 min at room temperature in deionized water and treated with 10% goat serum solution in 0.05% Tween 20—PBS was performed at room temperature for 1.5 h. Immunostaining was performed overnight in a humidified chamber at +4 °C using primary anti-GFAP antibody (Rabbit polyclonal, ab7260, Abcam, Waltham, MA, USA, diluted 1:1000), followed by 1.5 h-long incubation with goat anti-rabbit IgG (H + L) highly cross-absorbed antibodies (A11011, Alexa Fluor™ 568, Invitogen™, Thermo Fisher Scientific Inc., Carlsbad, CA, USA, diluted 1:1000) at room temperature. Finally, to visualize DNA/nuclei, all slices were stained with DAPI (62248, Thermo Fisher Scientific Inc., Carlsbad, CA, USA, diluted 1:1000) for 5 min and embedded with water-based Epredia™ Immu-Mount™ mounting medium (Thermo Fisher Scientific Inc., Kalamazoo, MI, USA).

### 2.6. Real-Time Polymerase Chain Reaction (qRT-PCR)

RNA was extracted using QIAzol and RNeasy Mini Kit (QIAGEN Sciences Inc., Germantown, MD, USA). cDNA was synthesized from 1 µg total RNA. SYBR (Bio-Rad Laboratories, Philadelphia, PA, USA) Green-based qRT-PCR was performed in 10 µL volumes. Primer sequences are listed in Table S1 (see Supplementary Materials); all primers were purchased from Life Technologies (Thermo Fisher Scientific Inc., Carlsbad, CA, USA). All samples were run in triplicate as described before [14,16,65].

### 2.7. Cell Culture Assay of Endotoxin-Induced IL-6 Release of Human Monocytes

The potency assay for NC was adapted from a previously established monocyte activation test [66]. Endotoxin-based potency assays are commonly used to assess the efficacy of anti-inflammatory agents, including stem cell-based therapies [67,68]. This method measures the release of IL-6 by human monocytes in response to endotoxin stimulation—a widely used translational marker of systemic inflammation [69]. Elevated IL-6 levels in LPS-stimulated monocyte cultures have been shown to correlate with the severity of both systemic and neuroinflammation [70].

A commercially available standardized human monocyte cell line was used (Merck, Darmstadt, Germany). Monocytes were exposed to reference standard endotoxin (RSE), in the absence of any further manipulations or following 15-h incubation with fresh NC at 37 °C, and 5% CO_2_, using manufacturer’s manual and standard reagents (Merck, Darmstadt, Germany). Protocol specifics were previously validated and controlled for appropriate the accuracy, specificity and linearity the potential effects of NC on IL-6 monocyte release in the absence of RSE was ruled out in control experiments.

### 2.8. Statistical Analysis

Data were analyzed using GraphPad Prism version 9.1.0 (San Diego, CA, USA). Shapiro–Wilk test was used as a normality test. For the data with normal distribution, three group comparisons were analyzed using ordinary one-way ANOVA followed by Holm–Šídák’s test when the variances were equal according to the Bartlett’s test, otherwise Welch’s ANOVA with post hoc Dunnett T3 test was applied. For the data that were not normally distributed, three-group comparisons were performed with Kruskal–Wallis test with post hoc Dunn’s test. For two-group comparisons with a normal distribution, unpaired Welch’s *t*-tests were used. For one-sample comparisons with random level, a one-sample *t*-test was applied for normally distributed results, and a one-sample Wilcoxon test was used for data that were not normally distributed. As we sought to confirm the validity of the APP/PS1 model and analyze the efficacy of NC in APP/PS1 mice, group comparisons were carried out only between groups differing by a single factor, i.e., WT vs. non-treated APP/PS1, and non-treated APP/PS1 vs. APP/PS1-NC. The level of significance was set at 95% (*p* < 0.05). Data were presented as mean ± SEM.

## 3. Results

### 3.1. Reduced Amyloid Plaque Formation and Attenuated Astrogliosis in Neuro-Cells-Treated APP/PS1 Mice

Representative images of amyloid plaques and astroglia from WT, untreated APP/PS1, and APP/PS1-NC mice are shown in Figure 2A. Brain sections were stained with Congo red to visualize amyloid plaques, and imaging was performed using a transmitted light detection module equipped with a photon-counting photomultiplier tube (PMT) for enhanced sensitivity. Figure 2B shows representative images of the hippocampal region stained with anti-GFAP (a marker of astrocytes) and counterstained with DAPI (a nuclear marker). Notably, NC-treated APP/PS1 mice exhibited reduced GFAP signal intensity and fewer GFAP-positive cells, suggesting attenuated astrogliosis.

**Figure 3:**
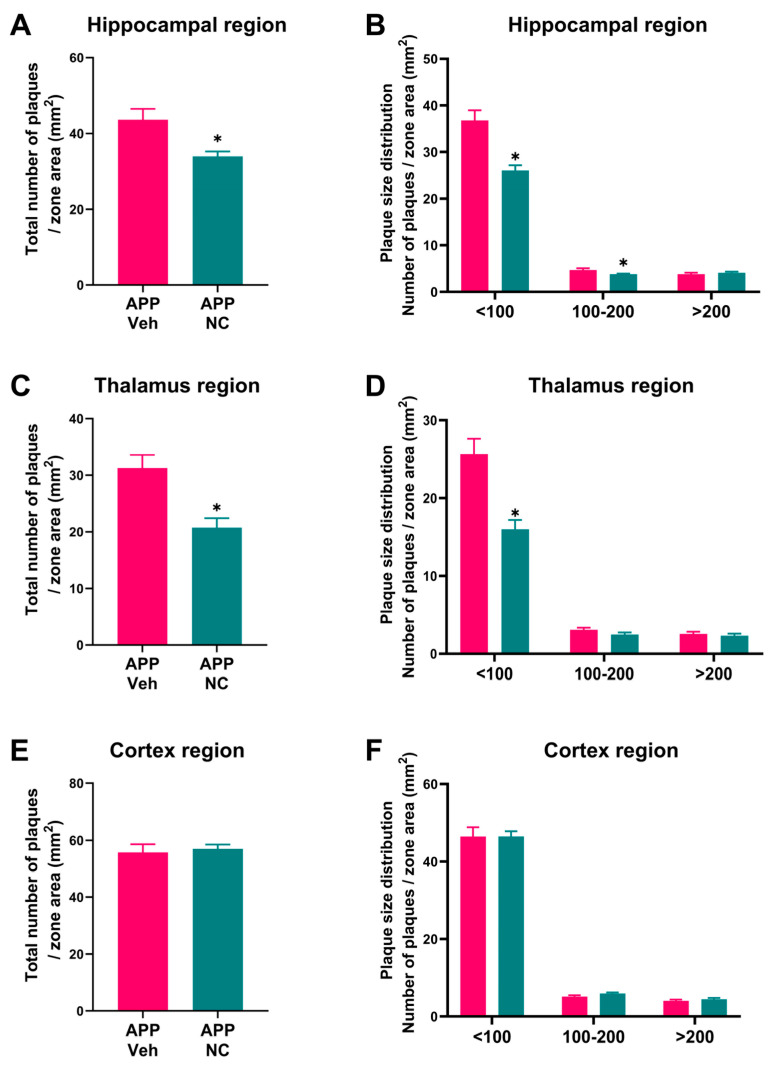
Quantitative analysis of amyloid plaques in the hippocampus, thalamus, and cortex regions of APP/PS1 and APP/PS1-NC mice. (**A**) The total number of amyloid plaques in the hippocampus of APP/PS1-NC and APP/PS1 mice. (**B**) Hippocampal amyloid plaques of various size categories. (**C**) The total number of amyloid plaques in the thalamus of APP/PS1-NC and APP/PS1 groups. (**D**) Thalamic plaque density analysis of various size categories in NC-treated and untreated mutants. (**E**) The total number of amyloid plaques in the cortex of APP/PS1-NC and APP/PS1 groups. (**F**) The cortical amyloid plaque density did not show significant differences in any size cat of various sizes. APP Veh = APP/PS1, APP NC = APP/PS1-NC. Data are presented as mean ± SEM. * *p* < 0.05, Welch’s *t*-test.

In the hippocampal region, the APP/PS1-NC group showed a significant reduction in the total number of amyloid plaques compared to the untreated APP/PS1 group, indicating a marked effect of NC treatment on plaque accumulation in this region (*p* = 0.0048, Welch’s *t*-test, Figure 3A).

When analyzing plaque density by size, significant differences were observed for plaques smaller than 100 µm^2^ (*p* = 0.00017) and for plaques between 100 and 200 µm^2^ (*p* = 0.03), but not for plaques larger than 200 µm^2^ (*p* = 0.50, Welch’s *t*-test, Figure 3B). In the thalamus, the total number of amyloid plaques in APP/PS1-NC mice was also significantly lower than in untreated APP/PS1 mice, reflecting the efficacy of NCs in reducing plaque burden in this brain region (*p* = 0.0011, Welch’s *t*-test, Figure 3C). Among plaque size categories, a significant reduction was noted only for plaques smaller than 100 µm^2^ (*p* = 0.0003), while no differences were observed for plaques sized 100–200 µm^2^ (*p* = 0.13) or larger than 200 µm^2^ (*p* = 0.56, Welch’s *t*-test, Figure 3D), suggesting a size-specific reduction effect. In the cortex, no significant difference in the total number of amyloid plaques was detected between the APP/PS1-NC and untreated APP/PS1 groups (*p* = 0.71, Welch’s *t*-test, Figure 3E) further indicating a region-specific limitation of NC treatment. Similarly, no significant differences were found when categorizing plaques by size (<100 µm^2^: *p* > 0.99, 100–200 µm^2^: *p* = 0.076, >200 µm^2^: *p* = 0.43, Welch’s *t*-test, Figure 3F).

### 3.2. Administration of Neuro-Cells Improves Memory and Reduces Anxiety-like Behavior in APP/PS1 Mice

**Figure 4:**
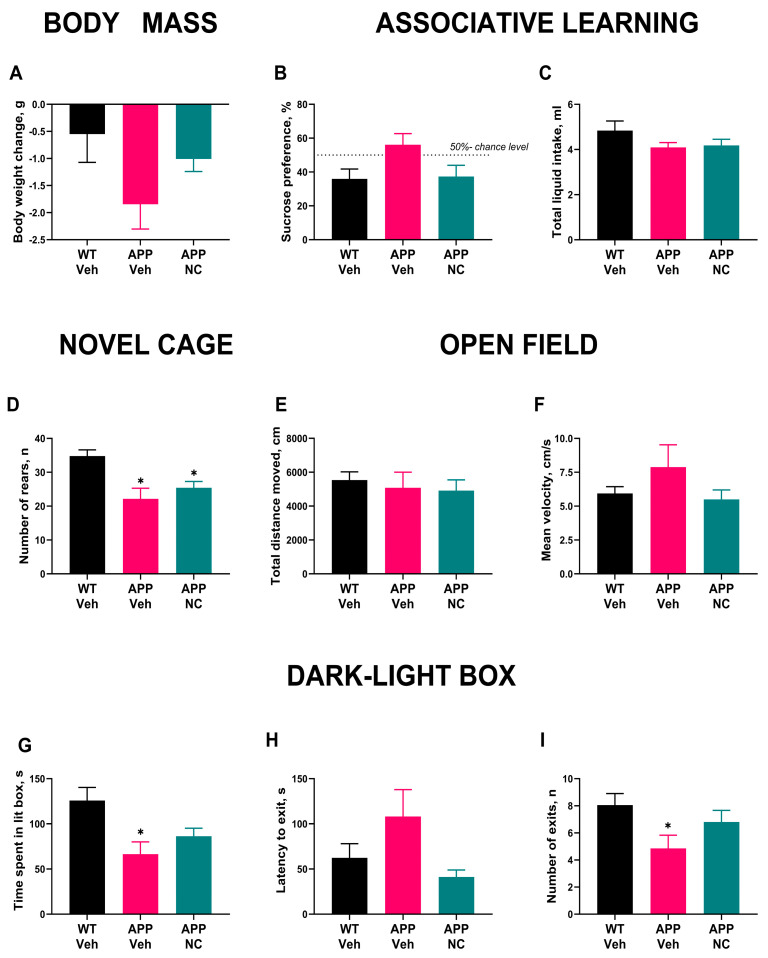
Behavioral changes in APP/PS1 mutants and effects of NC administration. (**A**) Body weight changes in APP/PS1 and APP/PS1-NC mice. (**B**) Acquisition of associative memory in the conditioned taste aversion test in WT, APP/PS1-NC and APP/PS1 groups of mice. (**C**) Total liquid intake in experimental groups of mice in the conditioned taste aversion test. (**D**) Rearing events in the novel cage test displayed by WT, APP/PS1 and APP/PS1-NC mice. (**E**) Total distance traveled and (**F**) mean velocity in the open field test scored in WT, APP/PS1 and APP/PS1-NC mice. (**G**) Time in the lit compartment of the dark–light box test spent by experimental groups of mice. (**H**) The latency to exit to the lit box shown by WT, APP/PS1 and APP/PS1-NC mice. (**I**) Number of exits to the lit compartment in WT, APP/PS1 and APP/PS1-NC groups of WT Veh = WT, APP Veh = APP/PS1, APP-NC = APP/PS1-NC. Statistical significance was evaluated as indicated for each test (see MS text) with * *p* < 0.05 vs. WT, * *p* < 0.05 vs. random level (50%). All data are presented as mean ± SEM.

Body weight changes over the experimental period did not significantly differ between groups, although the APP/PS1 group showed a non-significant trend toward lower body mass (F_2,29_ = 2.474, *p* = 0.1019, Figure 4A). These data indicate that NC administration did not significantly affect body weight, and any weight differences did not compromise the behavioral assessment of the experimental groups.

APP/PS1 mice exhibited memory deficits and increased anxiety, which were mitigated by the administration of Neuro-Cells. In the conditioned taste aversion test, WT-Veh mice showed significantly reduced sucrose preference compared to random (*p* = 0.042, one-sample Wilcoxon test), indicating successful learning of the association between sucrose and nausea (Figure 4B). In contrast, the APP/PS1-NC group did not exhibit a significant difference from the random level (*p* = 0.84), although there was a non-significant trend toward reduced sucrose preference (*p* = 0.1). These results suggest that NC treatment may partially restore associative learning in APP/PS1 mice. Total liquid intake did not differ significantly between groups (*p* = 0.39, Kruskal–Wallis test; Figure 4C), ruling out potential confounds related to fluid consumption in the assessment of associative learning. The absence of group differences in total intake indicates that the observed variation in sucrose preference during the recall session was not attributable to differences in drinking behavior or non-specific motivational factors.

In the novel cage test, both APP/PS1 and APP/PS1-NC mice showed reduced novelty exploration compared to WT Veh, as indicated by significantly fewer rears (*p* = 0.007 and *p* = 0.013, respectively, Kruskal–Wallis test with post hoc Dunn’s test, Figure 4D), indicating a reduction in exploratory behavior regardless of NC treatment. There was no significant difference between the APP/PS1 and APP/PS1-NC groups (*p* > 0.99). Additionally, the open field test showed no group differences in total distance traveled or mean velocity (F_2,28_ = 0.44, *p* = 0.65, and W_2 0,15 06_ = 0.86, *p* = 0.44, Figure 4E,F), suggesting that general locomotor activity was not impaired in APP/PS1 mice, and NC administration did not influence overall movement. As such, locomotor activity did not confound behavioral assessments in the study.

In the dark–light box test, the APP/PS1 group spent significantly less time in the lit box than WT mice (*p* = 0.015, Dunnett T3 test), reflecting increased anxiety-like behavior in these animals, while the APP/PS1-NC group did not differ significantly from WT mice (*p* = 0.078, Figure 4G), indicating a potential anxiolytic effect of NC treatment. Although the latency to exit the lit box was not significantly different (*p* = 0.26, Kruskal–Wallis test, Figure 4H), APP/PS1 mice showed a trend toward longer exit latency, suggesting that anxiety-like behavior was not fully normalized by NC treatment. The number of exits to the lit box was significantly lower in APP/PS1 mice than in WT mice (*p* = 0.04, Holm–Šídák’s test), further suggesting an increase in anxiety-like behavior in the mutants. Notably, this difference was not observed in the APP/PS1-NC group (*p* = 0.36, Figure 4I); this can be interpreted as a sign of decreased anxiety in this group of animals.

These results indicate that untreated APP/PS1 mice display anxiety-like behavior and memory impairment, while NC treatment reduces anxiety-like changes and partially restores cognitive performance.

### 3.3. Neuro-Cells Treatment Ameliorates Inflammatory and Cellular Plasticity Marker Expression in the Prefrontal Cortex of APP/PS1 Mice

**Figure 5:**
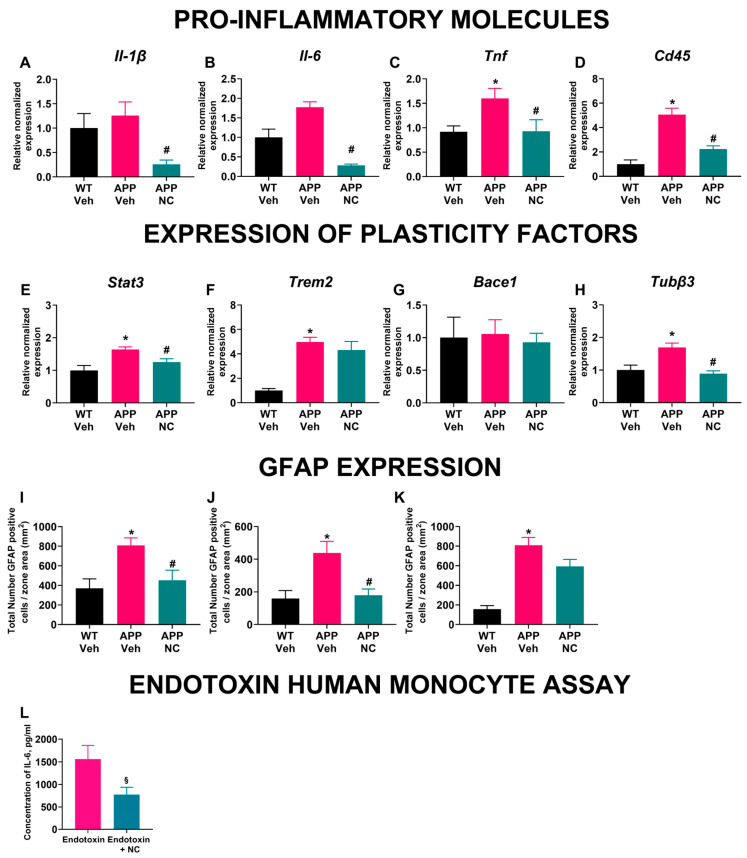
Anti-inflammatory effects of NC in vivo and in vitro. Gene expression in the prefrontal cortex of WT, APP/PS1 and APP/PS1-NC groups of (**A**) *Il-1β*, (**B**) *Il-6*, (**C**) *Tnf*, (**D**) *Cd45*, (**E**) *Stat3*, (**F**,**G**) *Bace1* and (**H**) *Tubβ3*. (**I**) The density of GFAP-positive cells in the hippocampus of WT, APP/PS1 and APP/PS1-NC mice. (**J**) The density of GFAP-positive cells in the thalamus of WT, APP/PS1 and APP/PS1-NC mice. (**K**) The density of GFAP-positive cells in the cortex of WT, APP/PS1 and APP/PS1-NC groups. (**L**) In vitro effects of NC pre-incubation on IL-6 release from endotoxin-stimulated human monocytes. WT Veh = WT, APP Veh = APP/PS1, APP-NC = APP/PS1-NC. Statistical significance was evaluated as indicated for each test with * *p* < 0.05 vs. WT, ^#^ *p* < 0.05 vs. APP/PS1, ^§^ *p* < 0.05 vs. Endotoxin group. All data are presented as mean ± SEM.

NC treatment significantly modulated the expression of inflammation and cellular plasticity markers in the prefrontal cortex of APP/PS1 mice. The gene expression of *Il-1β* showed significant group differences (F_2,14_ = 7.9, *p* = 0.005, Figure 5A), indicating that NC treatment can attenuate the inflammatory response. APP/PS1 mice exhibited a non-significant increase in *Il-1β* expression compared to WT, while NC-treated APP/PS1 mice showed a significant reduction compared to untreated mutants, suggesting that NC mitigates the inflammatory signaling cascade (*p* = 0.004, Holm–Šídák’s test). Similarly, *Il-6* expression differed significantly between groups (W_2 0,4 92_ = 50.88, *p* = 0.0005, Figure 5B). APP/PS1 mice showed a borderline increase compared to WT (*p* = 0.052, Dunnett’s T3 test), approaching statistical significance, while NC-treated APP/PS1 mice had significantly lower *Il-6* expression than untreated mutants (*p* = 0.0009). For *Tnf* expression, significant group differences were found (F_2,23_ = 4.27, *p* = 0.026, Figure 5C). APP/PS1 mice had significantly elevated Tnf compared to WT (*p* = 0.034), while NC treatment significantly lowered *Tnf* expression in APP/PS1 mice (*p* = 0.034), indicating a robust anti-inflammatory effect. The gene expression of *Cd45* also differed significantly between groups (F_2,14_ = 25.50, *p* < 0.0001, Figure 5D). APP/PS1 mice exhibited a marked increase compared to both WT (*p* < 0.0001) and NC-treated APP/PS1 mice (*p* < 0.0001).

*Stat3* expression was significantly elevated in the APP/PS1 group compared to both WT and NC-treated APP/PS1 mice (F_2,15_ = 5.640, *p* = 0.015; *p* = 0.009 and *p* = 0.049, respectively, Holm–Šídák’s test, Figure 5E) suggesting that NC treatment may attenuate the activation of the JAK/STAT3 pathway. For *Trem2*, significant differences were detected between groups (W_2 0,8 24_ = 48.42, *p* < 0.0001, Figure 5F). APP/PS1 mice had significantly higher *Trem2* expression compared to WT (*p* = 0.0004), but there was no significant difference between NC-treated and untreated APP/PS1 mice (*p* = 0.67), indicating that NC treatment did not modulate this specific inflammatory pathway. No significant group differences were observed in the expression of *Bace1* (F_2,16_ = 0.085, *p* = 0.92, Figure 5G), suggesting that NC treatment does not directly impact this enzyme’s transcriptional regulation. Lastly, *Tubβ3* expression was significantly higher in APP/PS1 mice compared to both WT and NC-treated APP/PS1 groups (F_2,16_ = 11.82, *p* = 0.0007; *p* = 0.0016 and *p* = 0.0005, respectively, Holm–Šídák’s test, Figure 5H). The normalization of Tubβ3 expression in NC-treated mutants suggests a possible stabilizing effect on cytoskeletal dynamics. These results indicate that NC treatment effectively reduces the expression of key inflammatory markers in the prefrontal cortex of APP/PS1 mice, suggesting a potential anti-inflammatory mechanism underlying the therapeutic effects of Neuro-Cells.

### 3.4. The Density of GFAP-Positive Cells Was Decreased in Neuro-Cells-Treated APP/PS1 Mice

In APP/PS1 mice, the density of GFAP-positive cells, an indicator of astrocyte activation, was significantly higher in the hippocampal and thalamus regions than in WT mice (*p* = 0.006 and *p* = 0.008, respectively; one-way ANOVA with post hoc Holm–Šídák’s test, Figure 5I,J), demonstrating pro-inflammatory changes and induction of astrogliosis. This increase was also significantly reduced in NC-treated APP/PS1 mice compared with in the untreated mutants (*p* = 0.017 and *p* = 0.013, respectively), indicating that NC treatment can reduce astrogliosis. In the cortex, untreated APP/PS1 mice displayed a significant increase in GFAP-positive cell density compared to WT mice (*p* < 0.0001, one-way ANOVA with post hoc Holm–Šídák’s test, Figure 5K). In contrast, NC-treated APP/PS1 mice showed a trend towards reduced GFAP expression compared to untreated APP/PS1 mice (*p* = 0.054), suggesting a partial attenuation of astrogliosis. These findings indicate that astrocyte activation, marked by increased GFAP expression, is elevated in APP/PS1 mice, but is markedly reduced following NC treatment. Thus, NC treatment led to a reduction in GFAP density compared to non-treated mutants.

### 3.5. Incubation with Neuro-Cells Diminished Endotoxin-Induced IL-6 Release of Human Monocytes

Human monocytes stimulated with endotoxin exhibited a substantial increase in IL-6 release into the media (Figure 5L). Pre-incubation with Neuro-Cells significantly reduced this endotoxin-induced IL-6 secretion (*p* < 0.0001, unpaired *t*-test) providing further evidence for the anti-inflammatory action of NC. These results demonstrate that NC exerts a pronounced anti-inflammatory effect on activated human monocytes in vitro.

## 4. Discussion

Our study demonstrates that the novel stem cell preparation, NC, significantly mitigate experimental AD-like syndrome in APP/PS1 mice. A single intracisternal infusion of NC significantly reduced amyloid plaque accumulation, improved cognitive and exploratory behavior, and decreased anxiety-like changes. These behavioral improvements were accompanied by a reduction in brain inflammatory markers and astrocytosis, highlighting the potential anti-inflammatory role of NC. Furthermore, the anti-inflammatory effects observed in vivo were corroborated by in vitro findings, where NC significantly reduced endotoxin-induced IL-6 release from human monocytes. Our results align with previous studies that reported the beneficial functional and anti-inflammatory effects of NC in a rat model of spinal cord injury [47] and in FUS-tg mice, a model of ALS/FTLD pathology [14,16]. Taken together, our findings suggest that NC may hold therapeutic potential for mitigating neuroinflammation and related neuropathological changes in AD. One of the key findings of our study was the significant reduction in amyloid plaque density in the hippocampal and thalamic regions of APP/PS1 mice treated with NC compared with untreated mutants. Notably, this reduction was primarily observed in the smallest plaques (<100 μm^2^), whereas the density of larger plaques (100–200 μm^2^ and >200 μm^2^) remained unchanged. Given the six-week period between NC administration and culling—ample time for new plaque formation [71]—it is likely that NCs inhibited the formation of new plaques rather than promoting the resorption of existing ones. This interpretation is supported by the selective reduction in smaller plaque sizes, suggesting that NC may specifically target the early stages of amyloid aggregation. The molecular changes observed in the brains of APP/PS1-NC mice support the hypothesis that NC treatment exerts therapeutic effects by modulating neuroinflammation and amyloid metabolism.

In particular, NC-treated mutants exhibited ameliorated expression of pro-inflammatory cytokines *Il-1β*, *Tnf*, and *Il-6*. These cytokines have been implicated in promoting Aβ accumulation [72], suggesting that the observed effects on their expression may underlie the positive impact of NC therapy on amyloid plaque formation. These findings are consistent with recent data showing that MSC-based therapies can selectively suppress pro-inflammatory cytokines, such as IL-1β, IL-6, and TNF, as well as the leukocyte marker CD45, through inhibition of the NF-κB and NLRP3 inflammasome pathways [73,74]. MSCs have been reported to secrete immunoregulatory factors, including prostaglandin E_2_ (PGE_2_), transforming growth factor-β (TGF-β), and IL-10, which inhibit NF-κB activation in macrophages and microglia. This leads to reduced expression of IL-6, TNF, and CD45, ultimately dampening immune cell activation [73,74]. Additionally, MSC-derived factors, such as PGE_2_ and stanniocalcin-1 (STC1), suppress mitochondrial reactive oxygen species (ROS) production and prevent TAK1/NF-κB-dependent priming of the NLRP3 inflammasome. As a result, inflammasome assembly, caspase-1 activation, and IL-1β maturation are inhibited [73,74]. Both in vitro and in vivo studies have demonstrated that MSC treatment reduces nuclear translocation of p65 and decreases the expression of NLRP3, ASC, and active caspase-1, leading to lower levels of IL-1β and TNF [73,75]. These immunomodulatory effects also promote the reprogramming of innate immune cells toward an anti-inflammatory M2 phenotype, characterized by elevated IL-10 and arginase-1 (Arg1) levels, reduced CD45^+^ leukocyte infiltration, and attenuated glial reactivity in models of inflammation and neurodegeneration [73,75]—effects likely mirrored in the present study using NC.

Indeed, stem cell-based therapies, particularly those employing MSCs, have consistently demonstrated potent anti-inflammatory actions by promoting the expression of tissue-repair and anti-inflammatory markers, such as IL-10, Arg1, and chitinase-like protein 3 (Ym1/Chi3l3). This shift reflects polarization toward an M2-like macrophage phenotype and resolution of inflammation [76,77]. The rebalancing of cytokine profiles is mediated by paracrine signaling and direct cell–cell interactions between stem cells and innate immune cells, including microglia and macrophages, altering their activation states [78]. In preclinical models of neuroinflammation and neurodegeneration—including Alzheimer’s disease and traumatic brain injury—MSC administration has been shown to reduce neuroinflammatory responses, suppress glial activation, and improve functional outcomes, effects largely attributed to these immunoregulatory mechanisms [79]. Thus, the coordinated downregulation of pro-inflammatory cytokines and upregulation of anti-inflammatory mediators represents a central mechanism underlying the therapeutic efficacy of stem cell interventions and is likely to contribute to the beneficial effects observed with NC in this study.

Moreover, our study revealed that NC administration significantly reduced the expression of *Stat3*, a key transcription factor linked to the JAK/STAT3 signaling pathway. As discussed above, this pathway is closely associated with the overexpression of pro-inflammatory cytokines and Aβ, which promotes reactive astrogliosis and Aβ-mediated neurotoxicity [4,18]. STAT3 hyperactivation has been linked to cognitive deficits in AD [4]. The effect reported in our study is consistent with previous data showing that MSC administration reduces phosphorylated STAT3 (pSTAT3) levels in affected brain regions, thereby limiting the transcriptional activation of pro-inflammatory genes [80]. It was proposed that this inhibitory effect may occur through secretion of anti-inflammatory mediators, such as TGF-β, PGE2, and extracellular vesicles containing microRNAs, including miR-124, miR-21, which interfere with upstream activators of the JAK/STAT pathway [81], as the stem cells of various origins were shown to secrete neurotrophins, anti-inflammatory factors and extracellular vesicles [41,42].

Consequently, the observed reduction in *Stat3* expression likely contributes to the attenuated astrogliosis detected in NC-treated APP/PS1 mice, which is particularly evident from the decreased density of GFAP-positive cells in the hippocampal and thalamic regions. This decrease aligns with a marked reduction in amyloid plaques in these areas, suggesting a region-specific therapeutic effect of NC on neuroinflammation and plaque pathology. In contrast, the cortical region showed less pronounced changes in GFAP expression and amyloid plaque density, indicating potential regional differences in the efficacy of NC treatment. These regional differences are likely attributable to several factors. First, delivery of stem cells into the cisterna magna has been shown to result in preferential distribution to periventricular brain regions, such as the hippocampus and thalamus. These areas are in closer proximity to cerebrospinal fluid (CSF) pathways and benefit from more robust glymphatic clearance mechanisms [82]. This may explain the significant reduction in astrogliosis and Aβ plaque deposition observed in the hippocampus and thalamus, but not in the cortex, of APP/PS1 mice treated with Neuro-Cells.

Supporting this, a previous study using the same mouse model demonstrated that MSC transplantation into the cisterna magna led to a marked decrease in the number and size of pyroglutamate-modified Aβ plaques in the hippocampus. This was accompanied by reduced expression of local IL-6 and TNF and a decrease in microglial activation, while these effects were far less pronounced in the cortex [83]. It has been suggested that this regional disparity is due to the limited diffusion of CSF-borne MSC secretome to superficial cortical regions, alongside regional variation in glymphatic inflow [82].

Moreover, stem cell treatment has been shown to downregulate inflammatory mediators and upregulate markers of microglial homeostasis predominantly in the hippocampus, but not in the cortex. These findings further support the presence of a region-specific immunomodulatory response to stem cell therapy, which may underlie the differential effects on plaque burden observed across brain regions [84].

Interestingly, our data also showed upregulation of *Tubβ3* expression in naive APP/PS1 mice, which was restored following NC treatment. *Tubβ3* is a microtubule protein critical for maintaining neuronal integrity and cytoskeletal dynamics, including vesicular transport and cell motility [20]. Dysregulation of microtubule-associated proteins has been reported in AD and is associated with disease progression and resistance to therapeutic interventions [20]. In preclinical models of spinal cord injury, transplantation of MSCs was shown to increase TUBΒ3 levels, concomitant with a significant reduction in inflammatory markers COX-2, IL-6, TNF, and oxidative stress indicators, such as 3-nitrotyrosine and 4-HNE [85]. Similarly, transplantation of neural stem cells or stem cell-derived exosomes in AD models promoted the maturation of neuronal phenotypes that were associated with enhanced TUBΒ3-immunoreactivity and restored neuronal protein distribution linked to improved synaptic and mitochondrial function [75]. It can be hypothesized that, similar to STAT, the triggering mechanism of action of NC is based on the secretion of anti-inflammatory factors and neurotrophines. The involvement of TUBΒ3 in the effects of stem cell therapy, as in the present study, indicates that this therapy promotes neuronal differentiation and axonal integrity. Thus, normalization of *Tubβ3* expression in NC-treated mice suggests that NC therapy may support cytoskeletal stability and neuronal function.

Another significant finding was the modulation of *Cd45* expression. Elevated *Cd45* levels, indicative of heightened immune responses, were detected in untreated APP/PS1 mice, consistent with neuroinflammatory changes in AD [86]. NC administration completely abolished this elevation, indicating a robust immunomodulatory effect. In contrast, the expression of *Trem2*, a receptor implicated in the regulation of IL-6, TNF, and Aβ metabolism [87,88], remained elevated regardless of treatment. This suggests that, while NC treatment can mitigate some inflammatory pathways, it may not fully counteract TREM2-associated mechanisms, which could be related to persistent innate immune activation or lipid dysregulation. Interestingly, the gene expression of *Bace1*, a major contributor to Aβ generation [89], was not significantly different between APP/PS1 and wild-type mice. This observation suggests that the therapeutic effects of NC on Aβ pathology may not involve direct modulation of BACE1 expression but might instead be mediated through downstream effects on inflammation or post-translational regulation [90,91].

As mentioned above, the anti-inflammatory effects of NC administered in vivo are consistent with the results of potency assays showing that NC reduced endotoxin-induced IL-6 release from human monocytes. Since reductions in IL-6 levels in a potency assay following exposure to stem cell preparations correlate with downregulation of TNF and IL-1β and are a shift toward anti-inflammatory phenotypes marked by increased IL-10 and Arg1 expression [68,92], these findings indicate broader relevance to immune reprogramming following NC administration. However, the in vitro panel used in our study was unable to investigate the release of other cytokines, which is a limitation of the current experiment.

The reduction in amyloid plaque density, mitigated neuroinflammation, and decreased astrogliosis observed in NC-treated APP/PS1 mice likely underpins the observed behavioral improvements. Behavioral analysis revealed that untreated APP/PS1 mice exhibited disrupted associative learning, increased anxiety-like behavior, as evidenced by reduced exploration in the dark–light box and novelty-suppressed exploration in the novel cage paradigm. In contrast, NC-treated mutants demonstrated normalized recall in the conditioned taste aversion paradigm, exploratory behaviors, indicating the potential anxiolytic effects of NC therapy. In models of Alzheimer’s disease, such as transgenic APP/PS1 mice or Aβ-injected rats, conditioned taste aversion performance is often impaired, reflecting hippocampus-dependent deficits in associative learning and aversive memory processing [93]. The hippocampus is critical for contextual and temporal modulation of aversive memory and for long-term retention and extinction of the taste-malaise association [94,95]. These impairments are typically associated with reduced hippocampal synaptic plasticity, neuroinflammation, and amyloid-beta pathology. Furthermore, the conditioned taste aversion paradigm has been employed to evaluate the efficacy of therapeutic agents aimed at restoring hippocampal function and memory performance in AD models [93].

The absence of changes in general locomotion, as assessed by the mean velocity and distance covered in the open field test, rules out non-specific motor effects as confounders. The amelioration of anxiety-like behavior in NC-treated mice could be attributed to a reduction in neuroinflammatory markers and amyloid burden, as previous studies have shown a close association between neuroinflammation, plaque pathology, and behavioral deficits in AD [96,97]. The relationship between anxiety and AD pathology is particularly relevant, as heightened anxiety is commonly observed in patients with AD and can significantly impact their quality of life [3]. In our study, NC treatment did not affect neophobic behavior in APP/PS1 mice, as assessed by the novel cage test. This may be explained by the fact that, although closely related, anxiety and neophobia represent distinct emotional responses to environmental challenges in mice, with overlapping but separable neurobiological underpinnings [98]. It is possible that NC therapy primarily targets neural circuits and mechanisms less involved in mediating the specific neurophysiological responses underlying neophobia in APP/PS1 mice.

Our findings align with those of previous reports demonstrating the therapeutic potential of stem cell-based interventions in AD models. Similar to our results, studies using MSCs have reported reduced Aβ deposition, enhanced plaque clearance, reduced neuronal apoptosis, and improved cognitive outcomes [99,100,101]. The underlying mechanisms are thought to include anti-inflammatory effects, reduction of hyperphosphorylated tau, and modulation of microglial response [40]. Intriguingly, recent preclinical models using intrathecal MSC transplantation have shown functional improvements, emphasizing the role of localized delivery in achieving therapeutic efficacy. Our results indicate that NC treatment may represent a promising strategy for mitigating neuroinflammation and behavioral deficits in AD. However, it remains to be determined whether these effects are sustained over longer periods and whether they translate into improved cognitive function in the advanced disease stages. Further studies are warranted to dissect the molecular pathways involved and assess the potential of NC as a therapeutic intervention for human AD.

## 5. Conclusions

Our study demonstrates that NC treatment exerts beneficial effects on key histological, behavioral, and molecular features of Alzheimer’s disease in the APP/PS1 mouse model. The observed reductions in amyloid plaque burden and astrogliosis, along with the normalization of pro-inflammatory cytokine expression, suggest that the therapeutic effects of NC are primarily mediated via anti-inflammatory mechanisms. Notably, improvements in anxiety-like behavior and exploratory activity were consistent with the attenuation of neuroinflammation and amyloid pathology. Although our primary aim was to identify transcriptional changes associated with NC treatment, given their potential to precede and predict protein-level effects, further validation at the protein level will be important to strengthen and extend these findings. Additionally, independent in vitro data showing that NC treatment reduces IL-6 secretion in endotoxin-stimulated human monocytes further supports its systemic anti-inflammatory potential. Together, these findings highlight NC therapy as a promising therapeutic candidate for AD and support further investigation into its long-term efficacy and translational applicability in clinical settings.

## Data Availability

Data available on reasonable request. To access data, Tatyana Strekalova (t.strekalova@pharm.ox.ac.uk and tatslova@gmail.com) should be contacted.

## Supplementary Materials

The following supporting information can be downloaded at: https://www.mdpi.com/article/10.3390/cells14151168/s1, Table S1. Cell populations in “Neuro-Cells” preparation. Table S2. Sequences for primers used in RT-PCR.

## Abbreviations

AD	Alzheimer’s disease
ALS	Amyotrophic lateral sclerosis
ANOVA	Analysis of Variance
APP	Amyloid precursor protein
Aβ	Amyloid beta
BACE1	Beta-secretase 1
BBB	Brain blood barrier
BDNF	Brain-derived neurotrophic factor
CD105+	Cluster of Differentiation 105 positive
CD271+	Cluster of Differentiation 271 positive
CD34+	Cluster of Differentiation 34 positive
CD45	Cluster of Differentiation 45
CD73+	Cluster of Differentiation 73 positive
CD90+	Cluster of Differentiation 90 positive
cDNA	Complementary DNA
CNS	Central Nervous System
DAPI	4′,6-diamidino-2-phenylindole
DNA	Deoxyribonucleic acid
FACs	Fluorescence-activated cell sorting
FDTL	Fronto-temporal dementia
GFAP	Glial fibrillary acidic protein
HGF	Hepatocyte growth factor
HSCs	Hematopoietic stem cells
i.c.	Intracerebral
i.c.v.	Intracerebroventricular
IGF-1	Insulin-like growth factor-1
IL-10	Interleukin-10
IL-1β	Interleukin-1 beta
IL-4	Interleukin-4
IL-6	Interleukin-6
JAK	Janus kinases
MSCs	Mesenchymal stem cells
NC	Neuro-Cells
NGF	Neuronal growth factor
PBS	Phosphate-buffered saline
qRT-PCR	Real-time quantitative reverse transcription polymerase chain reaction
RNA	Ribonucleic acid
RRR	Replacement, Reduction and Refinement
RSE	Reference standard endotoxin
SEM	Standard error of the mean
STAT3	Signal transducer of transcription factor-3
TNF	Tumor necrosis factor
T-PMT	Transmitted light photomultiplier
TREM2	Triggering receptor expressed on myeloid cells 2
Tubβ3	Tubulin beta-3 chain
VEGF	Vascular endothelial growth factor
WT	Wild type
