# Supplementary material for: Neuro-Cells Mitigate Amyloid Plaque Formation and Behavioral Deficits in the APPswe/PS1dE9 Model of Alzheimer Disease While Also Reducing IL-6 Production in Human Monocytes
Source: Cells. 2025 Jul 29;14(15):1168. doi: 10.3390/cells14151168 (PMC12345959; doi:10.3390/cells14151168)
Supplement: Supplementary file 1 [file cells-14-01168-s001.zip › cells-3700145-supplementary.pdf]

Supplementary Materials

Supplementary Table S1. Cell populations in "Neuro-Cells" preparation.

**Characterization of cell populations in "Neuro-Cells" preparation**	
**Cell populations in Neuro-Cells**	**Absolute number of cells injected per mouse in 10 μL**
Total number of nucleated cells	1.39×10^6/10 μL
**HSC**: total $CD34^+$ cells (calculated by FACs)	5.00×10^5/10 μL
Other mononucleated cells	8.9×10^5/10 μL
The proportion of MSCs expressing CD markers in overlapping manner (in total cell preparation, defined by single staining assay):	
**MSC**: $CD271^+$ cells (7%)	
**MSC**: $CD90^+$ cells (13%)	
**MSC**: $CD105^+$ cells (85.9%)	
**MSC**: $CD73^+$ cells (4%)	

Supplementary Table S2. Sequences for primers used in RT-PCR.

Gene	Name	Gene ID	Forward primer	Reverse primer
Gapdh	GAPDH	14433	ATGACCACAGTCCATGCCATC	GAGCTTCCCGTTCAGCTCTG
IL-1beta	IL-1beta	111343	TTGAAGTTGACGGACCCCAA	ATGTGCTGCTGCGAGATTTG
IL-6	IL-6	16193	TAGTCCTTCCTACCCCAATTTCC	TTGGTCCTTAGCCACTCCTTC
Ptprc	CD45	19264	CCTGCTCCTCAAACTTCGAC	GACACCTCTGTCGCCTTAGC
Tubb3	Beta 3-tubulin	22152	TGAGGCCTCCTCTCACAAGT	CATTGAGCTGACCAGGGAAT
Bace1	Bace1	23821	GTAACTTTGCAGTGGGGGCT	GCACATACACACCCTTTCGG
Trem2	Trem2	83433	ACAGCACCTCCAGGAATCAAG	ACTTGCTCAGGAGAACGCAG
Gfap	GFAP	14580	ACAGAGGAGTGGTATCGGTCT	GCGGCGATAGTCGTTAGCTT
Stat3	Stat3	20848	GACATTCCCAAGGAGGAGGC	CTTGGTCTTCAGGTACGGGG
Map2	Map2	17756	CTGGACATCAGCCTCACTCA	AATAGGTGCCCTGTGACCTG
